# Progressive improvement in time to diagnosis in axial spondyloarthritis through an integrated referral and education system

**DOI:** 10.1093/rap/rkae102

**Published:** 2024-08-23

**Authors:** Antoni Chan, Kathryn Rigler, Nadia Ahmad, Tanguy Lafont

**Affiliations:** University Department of Rheumatology, Royal Berkshire NHS Foundation Trust, Reading, UK; University Department of Rheumatology, Royal Berkshire NHS Foundation Trust, Reading, UK; University Department of Rheumatology, Royal Berkshire NHS Foundation Trust, Reading, UK; University Department of Rheumatology, Royal Berkshire NHS Foundation Trust, Reading, UK

**Keywords:** axial spondyloarthritis, diagnosis, primary care, referral pathways, secondary care, education

## Abstract

**Objectives:**

To assess the delay in the diagnosis of axial SpA (axSpA) in a real-world cohort over a 16-year period and to evaluate factors associated with this delay. We implemented a service improvement project and evaluated its effectiveness in improving time to diagnosis of axSpA.

**Methods:**

A cohort of axSpA patients newly diagnosed between January 2008 and December 2023 were studied. Surveys were carried out in 2013, 2017, 2019 and 2023 to assess time to diagnosis, which was divided into four periods from onset of inflammatory back pain to year of axSpA diagnosis. The time to diagnosis over the study period was analysed using a statistical process control chart.

**Results:**

Over the study period, 988 referrals were received and 366 (37%) had axSpA. There was a progressive increase in the number of females with axSpA. The mean time to diagnosis significantly decreased from 9.8 years (s.d. 1.2) in 2008 to 1.0 years (s.d. 1.0) in 2023. The greatest delay was from the onset of back pain to first seeing their general practitioners (GPs; mean 3.2 years). There was a significant improvement in the mean time to diagnosis across the time periods through the service improvement interventions.

**Conclusion:**

Structural and organizational change in triage, referral and clinic pathways has led to earlier recognition of axSpA. This is further enhanced through an integrated education program and awareness campaign for the public, GPs and healthcare professionals, including physiotherapists. With continuous quality improvement cycles, we achieved our aim of reducing the mean time to diagnosis to 1 year.

Key messages

- Time to diagnosis of axial SpA is reduced from a mean of 9.8 years to 1.0 year.
- Structural and organizational changes in the axSpA pathway were key to achieving this.
- An integrated education and training program upskilled the referrers for early identification of axSpA.

## Introduction

Axial spondyloarthritis (axSpA) is a chronic inflammatory disease characterized by inflammatory back pain, peripheral arthritis, enthesitis and extramusculoskeletal manifestations [1]. The delay in diagnosis of axSpA is 8.5 years on average in the UK [2] and is similar in other parts of the world [3].

Determinants of diagnostic delay in axSpA include female sex, negative HLA-B27 status, presence of psoriasis and younger age at symptom onset [4]. Delayed diagnosis may lead to higher morbidity from the condition [5, 6]. There have been several initiatives to reduce delays to diagnosis in axSpA [7]. These initiatives include increasing public awareness; improving referrals from primary care; increasing access to diagnostics, including MRI scans with axSpA protocols [8]; and working more closely with other specialties in secondary care. Referral pathways co-produced between primary and secondary care help promote timely diagnosis and treatment [9, 10]. The National Institute for Health and Care Excellence (NICE) in the UK has issued guidelines for SpA that aim to improve recognition and referral of patients with suspected axSpA [11]. This study evaluated the impact, challenges and effectiveness of a multifaceted approach implemented as a service improvement project at the Royal Berkshire Hospital to reduce the time to diagnosis in axSpA.

## Methods

This was a cross-sectional study of patients with newly diagnosed axSpA in a specialist rheumatology clinic. We studied a cohort of patients referred with back pain and suspected axSpA between January 2008 and December 2023. The study received institutional approval from the Royal Berkshire Hospital, Reading, UK (project N4484). As this was a non-interventional service improvement program, informed consent was not required. Paper case notes and electronic patient records, general practitioner (GP) records, physiotherapy records and pathology and radiology results were reviewed. A baseline survey (audit) was carried out from January to August 2013. Follow-up surveys were carried out from February to July 2017, January to May 2019 and October to December 2023.

**Figure 1.**
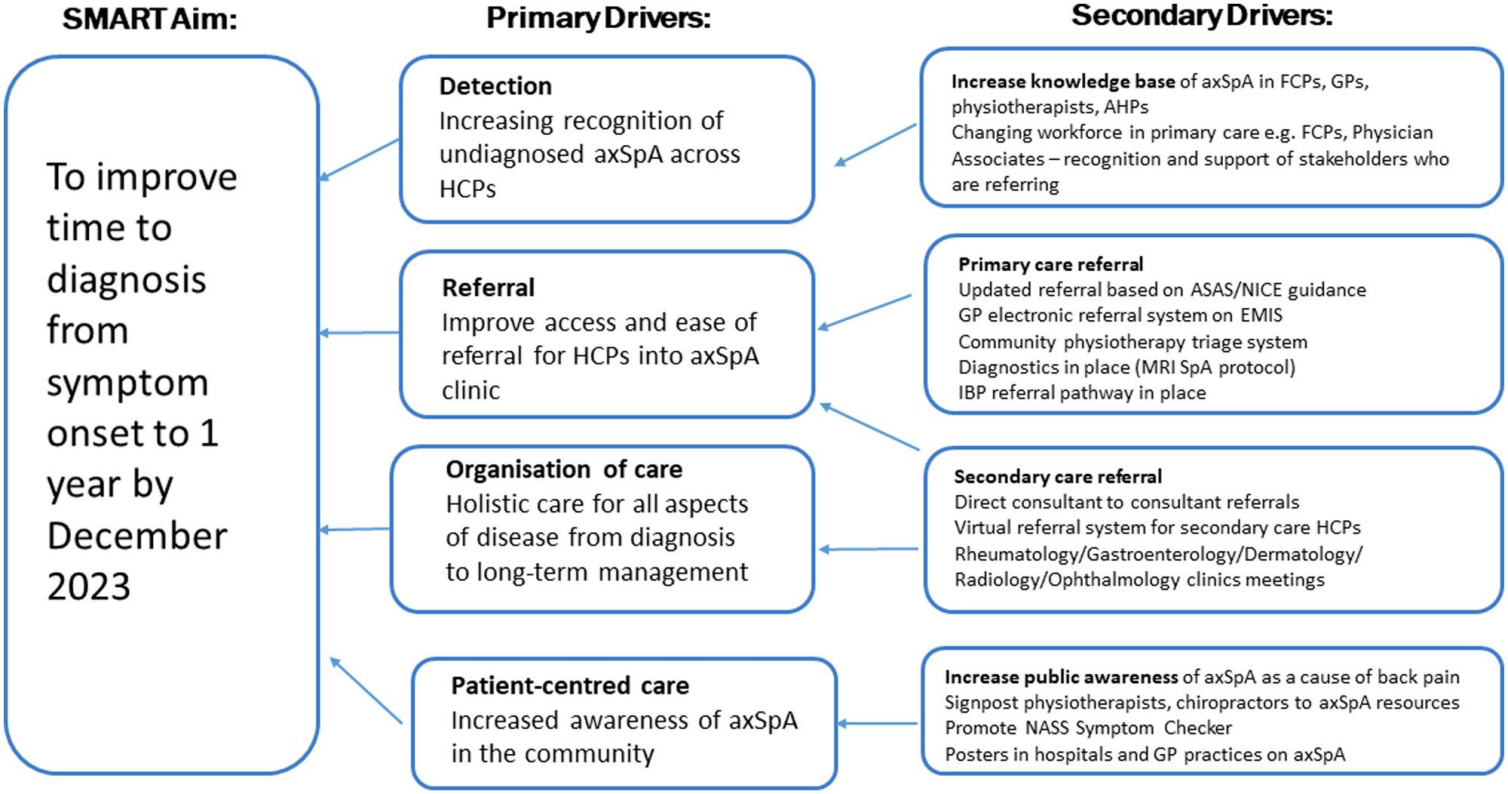
Driver diagram of specific, measurable, achievable, relevant, time-bound (SMART) aims of primary and secondary drivers to improve TTD of axSpA. FCPs: first-contact practitioners; AHPs: allied health professionals; EMIS: Egton Medical Information System; IBP: inflammatory back pain; NASS: National Axial Spondyloarthritis Society

The primary outcome measure was the time to diagnosis (TTD), calculated as the date between symptom onset of inflammatory back pain and the date of confirmed diagnosis of axSpA. A driver diagram was completed to evaluate the primary and secondary factors that affected TTD in axSpA (Fig. 1). The diagnosis of axSpA was clinical and confirmed by a consultant rheumatologist. All radiographs and MRI scans were reviewed and reported by five musculoskeletal radiologists in our centre. In cases of probable axSpA, cases were reviewed and discussed at the weekly Rheumatology–Radiology conference with attendance of a minimum of three consultant rheumatologists and two consultant radiologists. Consensus on the clinical diagnosis of axSpA was obtained for each case. Post-diagnosis classification was based on the Assessment of SpondyloArthritis international Society (ASAS) criteria for axSpA [12] or the modified New York criteria for AS [13]. We did not include patients with primary peripheral SpA or psoriatic arthritis with spondylitis.

The demographic, clinical and diagnostic variables were recorded. We analysed the trend, variation and changes in the durations over the 16-year study period using statistical process control (SPC), which is an analytical technique that plotted data over time, thus assisting us in understanding variation and guided us to take the most appropriate action. It also allowed any temporal patterns of fluctuations in the diagnostic journey of axSpA patients to be identified. The patient journey was divided into four distinct periods to analyse the time intervals and delays encountered in the diagnosis of axSpA. Period 1 was defined as the time from onset of back pain to the first visit to a GP, to capture the delay experienced by patients before seeking medical attention. Period 2 encompassed the duration from the GP visit to the referral to a rheumatologist, highlighting the time taken for appropriate referral. Period 3 represented the interval from the GP visit to the actual appointment in the rheumatology clinic, reflecting the waiting time within the healthcare system. Finally, period 4 measured the time from the rheumatology appointment to the formal diagnosis of axSpA, reflecting the time taken for diagnostics to be completed.

To compare the different periods, we employed statistical analysis to examine the mean durations and standard deviations. The *t*-test was used to compare two means and analysis of variance (ANOVA) was used to assess the significance of any differences observed between multiple means. The statistical analysis was conducted with a significance level set at *P* < 0.05 using R statistical software (version 4.3.3, R Foundation for Statistical Computing, Vienna, Austria). The various interventions used to reduce TTD are shown in Supplementary Table S1 and the referral form in Supplementary Fig. S1, both available at *Rheumatology Advances in Practice* online.

## Results

From 2008 to 2023, there were 966 referrals to the axSpA clinic for patients with back pain and suspected axSpA. From the referrals, 366 (37%) had a confirmed diagnosis of axSpA. The study flow diagram is shown in Supplementary Fig. S2, available at *Rheumatology Advances in Practice* online. In 622 patients (63%) the cause of back pain was not axSpA but was due to mechanical or other causes (Table 1). In a group of 103 patients (10.4%) who had an initial diagnosis of probable axSpA, through review at the multidisciplinary Rheumatology–Radiology meeting, 22 patients (2%) had axSpA and 81 (8%) did not have axSpA (not-axSpA).

**Table 1.** Demographics of patients in this study

Demographics	axSpA (*n* = 366)	Not-axSpA (*n* = 622)	All (*N* = 988)
Age at diagnosis, years, mean (range)	35.8 (17–59)	52 (16–89)	46 (16–89)
Male, *n* (%)	259 (71.0)	201 (32.3)	460 (46.6)
Ethnic background, *n* (%)			
White (including Eastern Europe)	334 (91)	505 (81)	839 (85)
Asian (including South and East Asia)	20 (5)	45 (7)	65 (7)
Hispanic	4 (1)	37 (6)	41 (4)
African	2 (1)	25 (4)	27 (3)
Other	6 (2)	10 (2)	16 (2)
Referral source, *n* (%)			
GP	261 (71.3)	473 (76)	734 (74)
Community physiotherapists	35 (9.5)	31 (5)	66 (7)
Specialist physiotherapist	12 (3.3)	12 (2)	24 (2)
Orthopaedics	29 (7.9)	75 (12)	104 (11)
Dermatology	9 (2.5)	19 (3)	28 (3)
Gastroenterology	10 (2.7)	6 (1)	16 (2)
Ophthalmology	10 (2.7)	6 (1)	16 (2)
Classification of axSpA, *n* (%)			
Radiographic axSpA	284 (78)	–	284 (29)
Non-radiographic axSpA	82 (22)	–	82 (8)
HLA-B27 positive, *n* (%)	328 (90)	93 (15)	421 (43)
CRP, mg/l, mean (s.d.)	5.8 (3.5)	5.4 (4.6)	5.6 (4.2)
X-ray SI joint positive for AS, *n* ( %)	284 (78)	–	284 (29)
MRI spine positive for axSpA, *n* (%)	342/351 (97)	–	342/792 (43)
MRI SI joint positive for axSpA, *n* (%)	190/351 (54)	–	190/792 (24)
ASAS classification of axSpA, *n* (%)	331 (90)	–	331 (34)
Extra-articular manifestations, *n* (%)	120 (33)	62 (10)	182 (18)
Psoriasis, *n* (%)	50 (14)	19 (3)	69 (7)
Uveitis, *n* (%)	55 (15)	31 (5)	86 (9)
Inflammatory bowel disease, *n* (%)	33 (9)	6 (1)	39 (4)
Ulcerative colitis, *n* (%)	13 (4)	4 (1)	17 (2)
Crohn’s disease, *n* (%)	20 (5)	2 (0.5)	22 (2)
Contact with other HCPs before diagnosis, *n* (%)			
Physiotherapist	255 (69)	267 (43)	522 (53)
Chiropractor/osteopath	71 (19)	236 (38)	307 (31)
Dermatologist	66 (18)	37 (6)	103 (10)
Orthopaedic	84 (23)	93 (15)	177 (18)
Ophthalmologist	55 (15)	56 (9)	111 (11)
Gastroenterologist	45 (12)	50 (8)	95 (10)
Diagnosis of not-axSpA patients, *n* (%)			
Mechanical back pain	–	404 (65)	404 (65)
SAPHO syndrome	–	22 (4)	22 (4)
Osteitis condensans ilii	–	32 (5)	32 (5)
Fibromyalgia	–	86 (14)	86 (14)
Polymyalgia rheumatica	–	48 (8)	48 (8)
Diffuse idiopathic skeletal hyperostosis	–	30 (5)	30 (5)

SAPHO: synovitis, acne, pustulosis, hyperostosis, osteitis.

The mean age of patients in the axSpA group was 39.5 years (range 17–59) and 47 years (range 16–69) in the not-axSpA group. The majority of axSpA patients were White [*n* = 334 (91%)]. The number of males with axSpA and not-axSpA were 259 (71%) and 201 (32%), respectively. The mean TTD in males was 6.3 years (s.d. 3.1) and in females was 5.0 years (s.d. 3.0). HLA-B27 positivity was 90% (238 patients) and 15% (93 patients) in the axSpA and not-axSpA groups, respectively. In the axSpA group, 284 (78%) were diagnosed as radiographic axSpA, meeting the modified New York criteria for AS [14]. There were 338 patients (92%) who met the ASAS classification criteria for axSpA [13]. In 351 patients who underwent MRI, the scan was positive in the SI joints in 342 (93%) and 190 (54%) in the spine.

**Figure 2.**
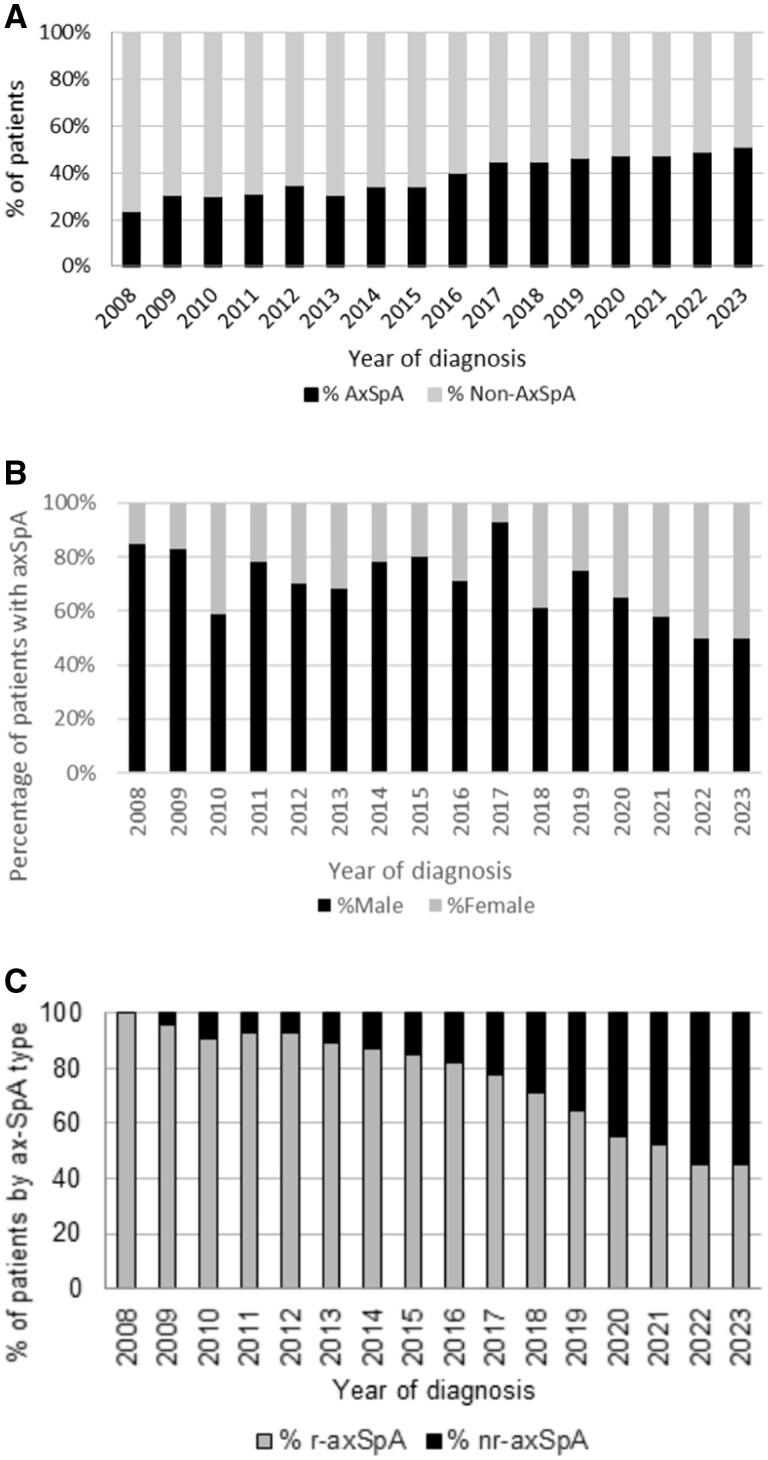
The number, sex and classification of axSpA in this study. **(A)** Percentage of patients referred with final diagnosis of axSpA (black-filled bars) and not-axSpA (grey-filled bars) by year of diagnosis. **(B)** The percentage of male patients (black-filled bars) and female patients (grey-filled bars) diagnosed with axSpA per year. **(C)** Percentage of radiographic axSpA (grey-filled bars) and non-radiographic axSpA (black-filled bars) in patients diagnosed with axSpA per year

The mean referrals per year for suspected axSpA was 73 (s.d. 10) for the period of 2008–2016 and 47 (s.d. 10) for 2017–2023. In 2008, the number of positive axSpA patients was 20 of 85 (23.5%) referrals and in 2023 this was 20 of 39 (51.3%) referrals (Fig. 2A). In the axSpA group, the referrals were from GPs [*n* = 261 (71.3%)], community physiotherapists [*n* = 35 (9.5%)], orthopaedics [*n* = 29 (7.9%)] and other specialties [*n* = 70 (19%)]. In 2008, the majority of patients with axSpA were males [*n* = 17 (85%)] and remained predominantly male until 2022, when the proportion of females increased to 50% (*n* = 11) (Fig. 2B). There were more patients with radiographic axSpA in the earlier years of diagnosis (100% in 2008) and slightly more of non-radiographic axSpA in the later years (55% in 2023) (Fig. 2C).

**Figure 3.**
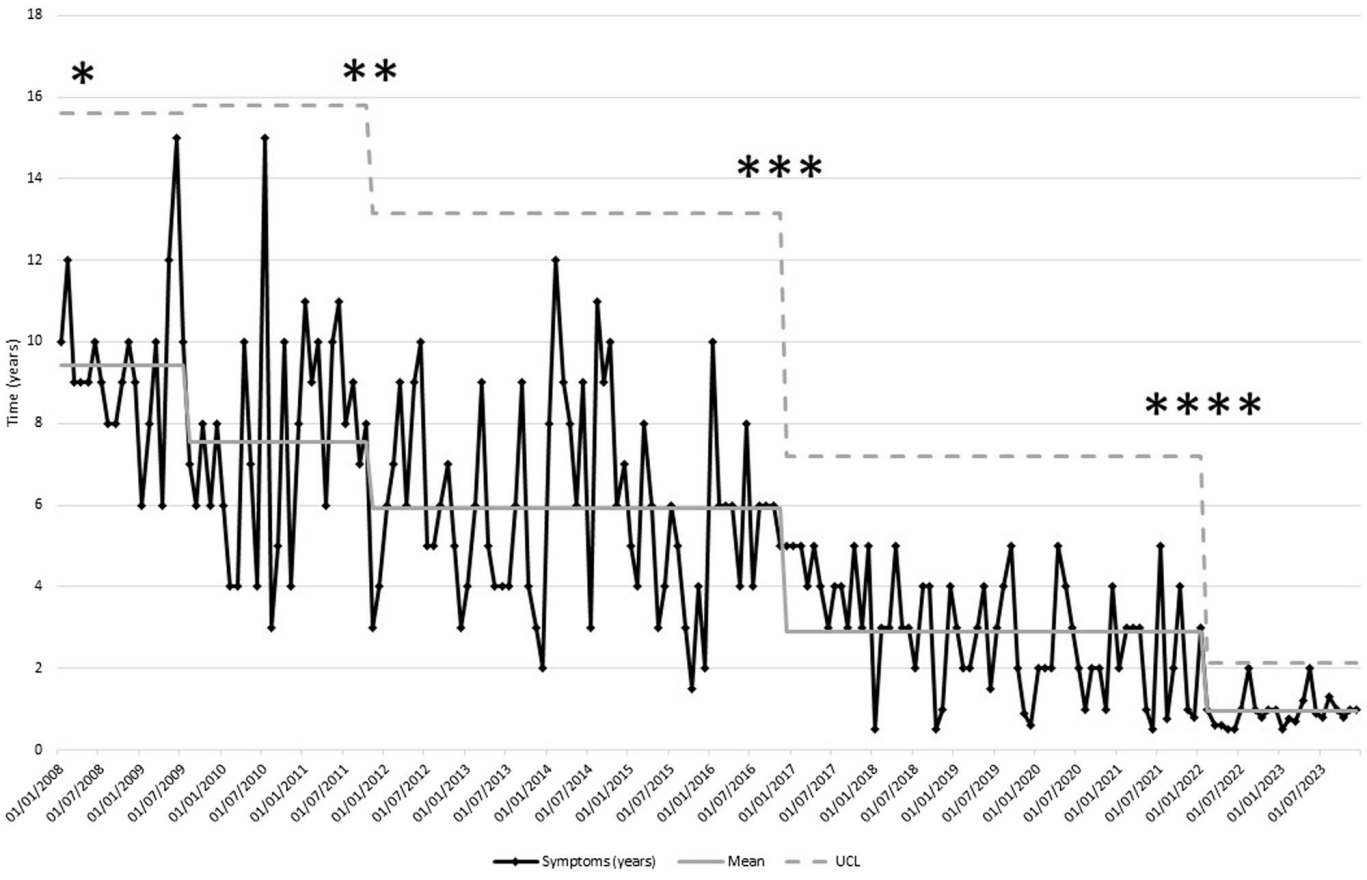
The SPC chart for the mean time to diagnosis (in years) of axSpA in the months during the period of the study from 2008 to 2023. *axSpA clinic/pathway and public awareness campaigns commenced. **Implementation and use of ASAS axSpA recommendations for referral [15]. ***Setting up of the IPASS. ****Implementation of the Rheumatology Academy and Collaborative Network (RheumACaN). *x*-axis: date of diagnosis (years); *y*-axis: time to diagnosis

The mean and median time to diagnosis significantly decreased from 9.8 years and 10.0 years (s.d. 1.2) in 2008 to 1.0 year and 1.0 year (s.d. 1.0) in 2023 (*P* < 0.05). The overall number of patients with a TTD of ≤2 years was 102 (28%). The TTD and reduction over time is shown in the SPC chart in Fig. 3. There were five mean times to diagnosis in years in the SPC: 9.4 (s.d. 2.1) in 2008–2009, 7.6 (s.d. 2.8) in 2009–2011, 5.9 (s.d. 2.4) in 2011–2016, 2.9 (s.d. 1.4) in 2017–2022 and 0.9 (s.d. 0.4) in 2022–2023. There was no lower control limit, as this goes below zero after the first mean of 9.4 years. There was a slight increase in the upper control limit (UCL) to 15.81 years on the second mean of 7.6 years. There was significant change in the UCL to 7.2 years as the time moved into the fourth mean of 2.9 years. The fifth mean was 0.9 years, which was below the target time to diagnosis of 1 year. The UCL was also significantly reduced in the final phase at 2.1 years. There was a significant difference observed between the means (*P* < 0.05).

**Figure 4.**
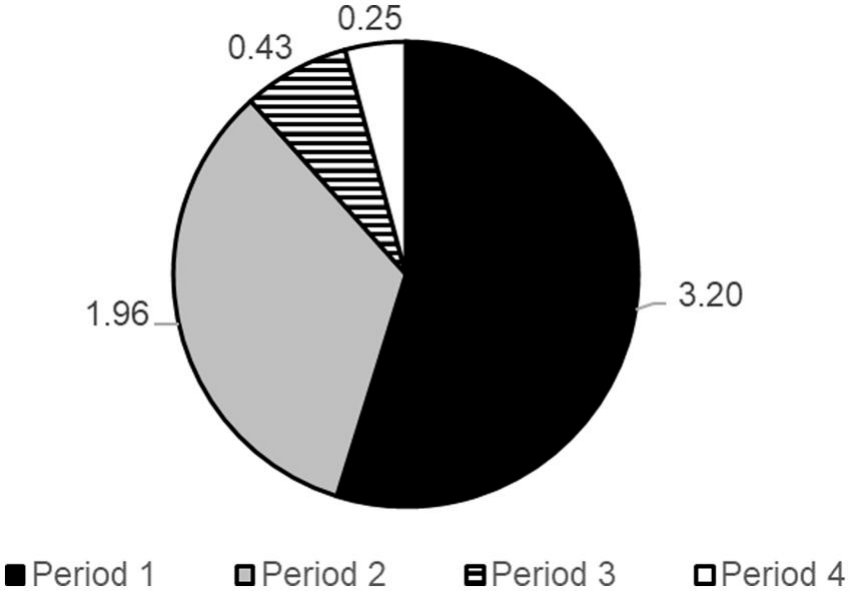
The mean time in years for each of the periods (1–4) during the study. Each period covers the time spent from symptom onset to visit to the GP (period 1), from seeing the GP to referral to a specialist (period 2), from referral to appointment with the specialist (period 3) and from appointment with the specialist to a formal diagnosis of axSpA (period 4)

**Figure 5.**
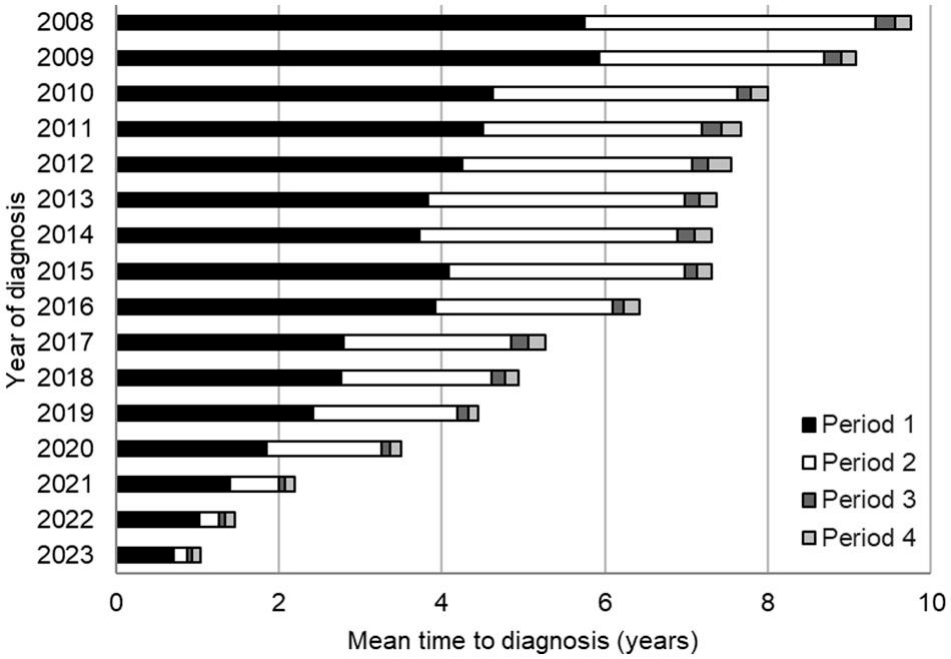
Time interval for each period (1–4) by year of diagnosis of axSpA. There was a significant difference between the means for each respective period (1–4) over the time of the study (*P* < 0.05)

The mean and percentage of the different periods (1–4) in the TTD from highest to lowest was in period 1 (3.2 years, 57.5%), period 2 (1.9 years, 36.7%), period 4 (0.3 years, 3.1%) and period 3 (0.4 years, 2.7%) (Fig. 4). The mean time intervals for four periods over the study period from 2008 to 2023 are shown in Fig. 5. There was a significant difference between the means for periods 1–4 during the study (*P* < 0.05). The reduction in the mean time interval for each period from 2008 to 2023 was −5.0 years in period 1, −3.4 years in period 2, −0.2 years in period 3 and −0.1 years in period 4.

## Discussion

AxSpA has a mean TTD of 6.7 years [16] and a median of up to 8 years [17]. The International Map of Axial Spondyloarthritis (IMAS) study showed a mean TTD of 7.4 years (s.d. 9.0) [18]. In our study, we significantly reduced the mean TTD from 9.8 years in 2008 to 1.0 year in 2023. This is in line with the national TTD target of 1 year [7]. The greatest delay was in period 1, which was 57% of the total TTD. In the first 4 years of the study, from 2008 to 2011 inclusive, there was a slight increase in the UCL on the second mean in the SPC (Fig. 3). During this period, we were identifying patients who already had symptoms for a long period of time and at the start of the program, where the mean was 9.42 years. This showed that while we were undertaking the improvement work, we still did not have a grasp of the outliers in terms of time to diagnosis of axSpA. This was probably because the public and HCPs were only starting to identify cases and referring them to our axSpA clinic. The effect of public and HCP awareness campaigns took time for symptoms to be noted. During this time we did not have an external group that was assessing and triaging patients in the community. We were also focused on our internal processes during this time, such as developing our diagnostic pathways such as axSpA MRI scan protocols.

Prior to the diagnosis of axSpA, there were contacts with physiotherapists in 69% and chiropractors in 19% of axSpA patients (Table 1). A higher number of HCPs seen before diagnosis was also associated with a greater delay [3]. A UK survey of the confidence of physiotherapists in recognizing and referring patients with suspected axSpA showed higher recognition of non-specific lower back and radicular pain compared with axSpA [19]. There are barriers in both the recognition and communication of symptoms of axSpA between both patients and HCPs that may increase TTD [20]. The setting up of the Integrated Pain Assessment and Spinal Service (IPASS) in 2016 resulted in more targeted referrals from 2017 to 2023 (Fig. 2A). There was an increase in the accuracy of referrals, with positively diagnosed patients increasing from around 1 in 5 (23.5%) at the start of the study to 1 in 2 (51.3%) at the end of the study. Physiotherapists can play a key role in triage, as the majority of patients (69%) were seen by them in period 1, prior to seeing their GPs. Physiotherapists also conducted the IPASS clinic where triage took place in the community, resulting in more accurate referrals and a reduction in TTD.

The TTD can be affected by the sex of the patient [21]. In 2008, the majority of axSpA patients (85%) were male. The proportion of females gradually increased to 50% in 2022 (Fig. 2B). The trend towards a male:female ratio of 1:1 fits with the ratio that is commonly cited now [22]. Our data are also supported by the epidemiological primary care data from England showing that the incidence of axSpA was equal in males and females in 2020 [23]. As the male:female ratio was equal after 2022 in our study, this may mean that there were female patients that were not diagnosed at the start of the study. Data collected from 2008 to 2015 reflect a time period when axSpA was still thought of in primary care as more dominant in males and this may have influenced referral patterns. As male patients are more likely to have radiographic change compared with females, it is highly likely that during this time period males contributed more radiographic axSpA in the earlier years of this study. Female patients show a higher diagnostic delay compared with males [24], and increased awareness of the sex differences in the manifestations of axSpA [25], by both clinicians and researchers, is needed for diagnosing axSpA early [26]. During our primary care education program, we discussed the lack of early MRI change in women and how they may present with a more chronic, widespread pain pattern of possible axSpA in females, aiding referral decisions and identification of axSpA in women [27]. This may have resulted in the non-significant difference of TTD in female axSpA patients with a mean of 5.0 years (s.d. 3.0) compared with 6.3 years (s.d. 3.1) in males. Another factor that could impact the male:female ratio in our study is that we specifically looked for axSpA rather than peripheral SpA, and women can often present with more peripheral symptoms [28].

Period 2 was the second-longest interval in the TTD, contributing to 37% of the total time. An integrated referral system was implemented in 2015. Referral strategies for early diagnosis of axSpA have led to the diagnosis of as many as 33–45% of patients within a target population with axSpA [29]. The electronic referral system was updated annually according to best practice guidelines and appeared as a decision support tool when patients were referred to the axSpA clinic. In the SPC, there was a significant change in the UCL as we moved into the fourth mean, suggesting that we had more control over this process and an improvement in referrals (Fig. 3A). GPs are another group of HCPs, in addition to physiotherapists, that play a key role in identifying patients with axSpA and subsequent early referral to reduce TTD.

The increased referral accuracy to 49–51% in 2022–2023 correlated with the implementation of the Rheumatology Academy and Collaborative Network (RheumACaN) in 2021 (Fig. 2A). In 2021 there was further improvement in the TTD. The fifth mean (0.95 years) was below the target of 1 year. The variation in the time to diagnosis (UCL) was significantly reduced (*P* < 0.05) (Fig. 3). This showed that we had a grip on both the GP referrals and the internal processes. These findings are in line with previously published evidence on the beneficial effects of GP education on awareness and recognition of axSpA [30]. The ASAS consensus definition of early axSpA is ≤2 years from symptom onset [31]. Using this definition, the number of axSpA patients in our study was 102 (28%), with more patients diagnosed in recent years meeting this definition.

Period 3 contributed 2.7% to the overall time to diagnosis, with a mean of 0.16 years (s.d. 8.3 weeks). There was an improvement from a baseline of 0.23 years (s.d. 11.9 weeks) to 0.06 years (s.d. 3.1 weeks) in 2023 (Fig. 4). We have implemented an early inflammatory arthritis pathway that includes axSpA as well as RA and PsA. Patients with suspected axSpA are seen promptly in the clinic with a target of 3 weeks from GP referral.

The overall mean for period 4 was 0.18 years (s.d. 9.4 weeks), which contributed to 3.1% of the overall TTD in the study. We implemented a standardized MRI protocol for suspected axSpA in 2015 [32]. This has helped to improve period 4 from 0.20 years (s.d. 10.4 weeks) in 2008 to 0.11 years (s.d. 5.72 weeks) in 2023. The reduction in TTD was not solely due to the increase in diagnosis of non-radiographic axSpA, as there was also a similar TTD reduction in the radiographic axSpA group (Fig. 2C). This highlights the equal burden of disease in both non-radiographic and radiographic axSpA [33].

Overdiagnosis of axSpA may occur with the use of classification criteria [34] and increased utilization of MRI scans [35]. In the evaluation at the time of referral, 103 patients (10%) had probable axSpA. This is similar to a previous study of patients with chronic low back pain from primary care, with 9.1% having possible axSpA [36]. In our centre, these patients were carefully evaluated in our Rheumatology–Radiology meeting, where the clinical and radiological features were assessed to reach a final consensus on the diagnosis of axSpA. This practice of combining clinical information and radiological findings has been shown to increase axSpA diagnostic accuracy [37]. Both the rheumatologists and radiologists attended training together on MRI interpretation to improve diagnostic confidence, which has improved diagnostic accuracy in axSpA imaging [38]. It has been shown that rheumatologists can unequivocally diagnose axSpA in a third of patients with chronic back pain of <2 years’ duration. HLA-B27 and MRI positivity at baseline discriminated best at the stable 2-year axSpA diagnosis [39].

Following diagnosis of axSpA, we applied the ASAS classification criteria and 331 (90%) patients met these criteria. There were 27 axSpA patients (7.3%) who had onset of symptoms after the age of 45 years. Our findings are in keeping with a global ASAS study that showed 92% of patients had onset of axial symptoms at <45 years [40]. Focusing on the imaging arm of the ASAS classification criteria, 9 axSpA patients (3%) did not have MRI sacroiliitis but did have inflammatory lesions consistent with axSpA in the spine [41]. The finding of isolated spinal inflammation on MRI in axSpA is supported by other studies [42, 43].

Since the inception of our specialist axSpA service in 2008 we have run many improvement and awareness initiatives to improve TTD. Creating change in the real world takes time and the improvement in TTD is a culmination of these initiatives (Supplementary Table S1, available at *Rheumatology Advances in Practice* online). However, as demonstrated in Fig. 3, there were two key initiatives that have had the biggest impact on reducing TTD. First, setting up the community triage service (IPASS) increased the referral to diagnosis conversion rate and accuracy of referrals. Second, implementation of the RheumACaN program with collaborative education and mentoring for primary care decreased the TTD further to the 1 year target. Improved confidence in recognizing and referring patients with suspected axSpA also ensured that patients who do not need referral to rheumatology are then seen more promptly in a more relevant service for them, reducing the economic burden of inaccurate referrals. These interventions can be implemented and integrated into current clinical practice. There are existing community musculoskeletal triage services where patients with back pain are assessed. Working with these services to identify axSpA and early referral from GPs are implementable. The clinical implication of our study is that collaboration and integration with community musculoskeletal services and primary care resulted in a reduction of the TTD.

The strength of this study is that it uses real-world data and time frames for analysis and implementation of quality improvement projects. Continuing usual clinical practice and caseloads while trying to implement quality improvement projects can create delays in implementation of projects or they take longer to come to fruition than would be ideal. However, this reflects usual practice across most hospitals and therefore this study demonstrates initiatives and improvements that are achievable within a normal hospital working environment. The limitations of this study included retrospective data collection from patient records, which impacts deeper analysis of some of the data. For example, we do not have follow-up data on patients referred between 2008 and 2015 who were not axSpA and so cannot discuss what condition they may have progressed into or referrals onwards from this point. We also did not collect follow-up data from axSpA patients to understand the progression of disease or the development of comorbidities. Our ethnicity data also has a large Caucasian group, so collecting data prospectively will allow us to identify further nuances in axSpA referral and diagnosis based on ethnicity. Our aim was to meet and maintain the National Axial Spondyloarthritis Society gold standard of no more than 1 year in the TTD of axSpA.

The significant reduction in the TTD of axSpA in this study is attributed to the implementation of various interventions with a quality improvement focus. These interventions included educating GPs to recognize early symptoms, facilitating electronic referrals for expedited specialist evaluation and implementing community physiotherapy triage and assessment services. The education of GPs is aimed at increasing awareness of axSpA symptoms and encouraging early referral to specialist rheumatology service. The utilization of electronic referrals streamlined the referral process, ensuring prompt assessment and diagnosis. Additionally, community physiotherapy triage and assessment services provided patients with early access to specialized care, aiding in the timely detection and diagnosis of axSpA. Further research is warranted to assess the long-term impact of these interventions on patient outcomes and to explore their applicability in other healthcare settings.

## Supplementary Material

rkae102_Supplementary_Data

## Data Availability

Data cannot be shared for privacy reasons. The data within the study is from health data for a population in a single location. Given the nature of the data and number of participants involved, it is not possible to provide a minimal de-identified dataset that retains the necessary data utility to replicate our study’s findings and be considered anonymized.
