# Supplementary material for: Progressive improvement in time to diagnosis in axial spondyloarthritis through an integrated referral and education system
Source: Rheumatol Adv Pract. 2024 Aug 23;8(4):rkae102. doi: 10.1093/rap/rkae102 (PMC11398972; doi:10.1093/rap/rkae102)

**Supplementary Table S1. Interventions implemented in the Axial Spondyloarthritis (axSpA) service to improve time to diagnosis**

| **Intervention** | **Summary of the intervention** | **Date of implementation** |
| --- | --- | --- |
| Setting up the specialist Axial Spondyloarthritis (axSpA) clinic in the hospital | Setting up of the weekly axSpA clinic to receive referrals and recording time to diagnosis and clinical outcomes | 2008 |
| Co-production of GP referral pathway for axSpA | Production and implementation of first version of the GP referral pathway to the axSpA service | 2008 |
| Public awareness campaign | Increased public awareness of axSpA using posters, stands, local newspapers, *Don’t Turn Your Back On It* campaign | 2009 |
| Primary care healthcare professional education | Annual educational events for primary care healthcare professionals including GPs to improve referrals for axSpA | 2010 |
| Standardised GP referral criteria | Implementation and use of ASAS inflammatory back pain criteria for GP referrals | 2011 |
| Early access to AxSpA MRI protocol scans | Standardisation of the MRI imaging protocols using the BRITSPA consensus MRI protocol | 2015 |
| Implementation of ASAS referral criteria | Use of the ASAS referral criteria for GP and community referrals | 2016 |
| Community triage service | Setting up of the Integrated Pain Assessment and Spinal Service (IPASS) for the triage and management of MSK conditions including axSpA | 2016 |
| Audit of axSpA service using the NICE guidelines on spondyloarthritis (NG65) | Audit of referral and time to diagnosis based on NICE guidelines on spondyloarthritis | 2017 |
| Electronic GP referral system and decision making tool | Integration of axSpA referral pathway and criteria into electronic GP system including decision making tool | 2018 |
| Access to early inflammatory arthritis (EIA) clinic slots for patients with suspected axSpA | Participation in the National Early Inflammatory Arthritis Audit (NEIAA) and including axSpA patients in EIA slots | 2019 |
| Selection and participation in the National Axial Spondyloarthritis Society (NASS) Aspiring to Excellence Programme | Selection into Cohort 1 of the NASS Aspiring to Excellence Programme with support from the NHS Transformation Unit using quality improvement methodology to improve time to diagnosis and the axSpA service | 2019 |
| Mentoring and support for GPs and community physiotherapists | Implementation of the Rheumatology Academy and Collaborative Network (RheumACaN) which provides mentoring for GPs and community physiotherapists | 2020 |
| Combined clinics with other AxSpA related specialities | Combined clinics with Dermatology, Gastroenterology and virtual MDT with Ophthalmology to improve detection of axSpA | 2021 |
| Completion of 3 cohorts of RheumACaN | Completion of 3 cohorts of RheumACaN. Working with primary care and the community to improve axSpA referrals. | 2023 |

**Supplementary Figure S1. The Royal Berkshire Hospital referral form for suspected axSpA.**

|  |
| --- |

| **Axial Spondylarthritis Referral Form**   - *Please use this form if you believe the patient requires rapid assessment of the symptoms / signs of inflammatory arthritis listed below. (Otherwise please refer the patient in the usual way for your practice)* - *Patients meeting criteria for suspected inflammatory arthritis with relevant investigations completed will be seen within 3 weeks of receipt of referral in line with national guidance.* |
| --- |

| ***Suspected Condition*** | *If Early Inflammatory Arthritis is suspected, please use the relating form* |  | *Axial Spondyloarthritis (Axial SpA) |  |
| --- | --- | --- | --- | --- |
| *If back pain is the main feature identified, please select the Axial Spondyloarthritis option | | | | |

| **Patient Details** |  |  |  |  |  |  |
| --- | --- | --- | --- | --- | --- | --- |
| *Surname:* |  | *DOB:* |  | | | |
| *First Name:* |  | *Hospital No. (if known)* |  | | | |
| *Address:* |  | | | | | |
| *Mobile No.:* |  | | | | | |
| *Evening Tel:* |  | *Day Time Tel.:* |  | | | |
| *First Language* |  | *Interpreter Required* | Yes |  | No |  |
| *Sex* |  | *Wheelchair Assistance* | Yes |  | No |  |

| **Indications of Axial Spondyloarthritis (Axial SpA)** | | | | | | |
| --- | --- | --- | --- | --- | --- | --- |
| Patient has had Chronic Back Pain for >3 Months with first symptom presenting <45 years of age | | | | | | |
| Yes |  | *If* ***no****, please consider other diagnosis for back pain* | | | | |
| **Features of Inflammatory Backpain** | | | **Select if present** | | |  |
| Improvement with exercise and worse with rest | | |  |  |  |  |
| Pain in second half of the night due to pain | | |  |  |  |  |
| Alternating buttock pain | | |  |  |  |  |
| Improvement within 48 hours of NSAIDs | | |  |  |  |  |
| **Associated Clinical Features** | | |  |  |  |  |
| Current or past arthritis | | |  |  |  |  |
| Current or past enthesitis | | |  |  |  |  |
| Current or past psoriasis | | |  |  |  |  |
| Current or past inflammatory bowel disease  (Crohn’s or ulcerative colitis) | | |  |  |  |  |
|  | | |  |  |  |  |
| **Family History** | | |  | | | |
| First Degree relative with spondyloarthritis | | |  |  |  |  |

| **Features and Family History Total**  *(3 or more required for referral)* | *Enter total here* |
| --- | --- |

| **If >3 features from above, investigate** with:** | | **Select if Completed:** | | | **Result** |
| --- | --- | --- | --- | --- | --- |
|  | |  | | | *Negative investigations do not exclude Axial SpA |
| HLA-B27 | |  |  |  |  |
|  | |  |  |  |  |
| FBC, ESR, U&E, LFT, CRP | |  |  |  | FBC |
|  | |  |  |  |  |
|  | |  |  |  | ESR |
|  | |  |  |  |  |
|  | |  |  |  | U&E |
|  | |  |  |  |  |
|  | |  |  |  | LFT |
|  | |  |  |  |  |
|  | |  |  |  | CRP |
|  | |  |  |  |  |
| **Please append additional relevant test results | |  |  |  |  |
| Please fill in relevant sections below in as much detail as possible | | | | | |
| Referring Notes: |  | | | | |
| Drug Allergies: |  | | | | |
| Medical Conditions: |  | | | | |
| Current Medications: |  | | | | |

| Clinician Signature: |  | Referral Date: |  |
| --- | --- | --- | --- |

| - Please refer to *Guidance for Healthcare Professionals Referring into Rheumatology* for full referral details - On Completion please attach in eRS to Rheumatology. |
| --- |

**Supplementary Figure S2. Study flow diagram of patients by diagnoses.**


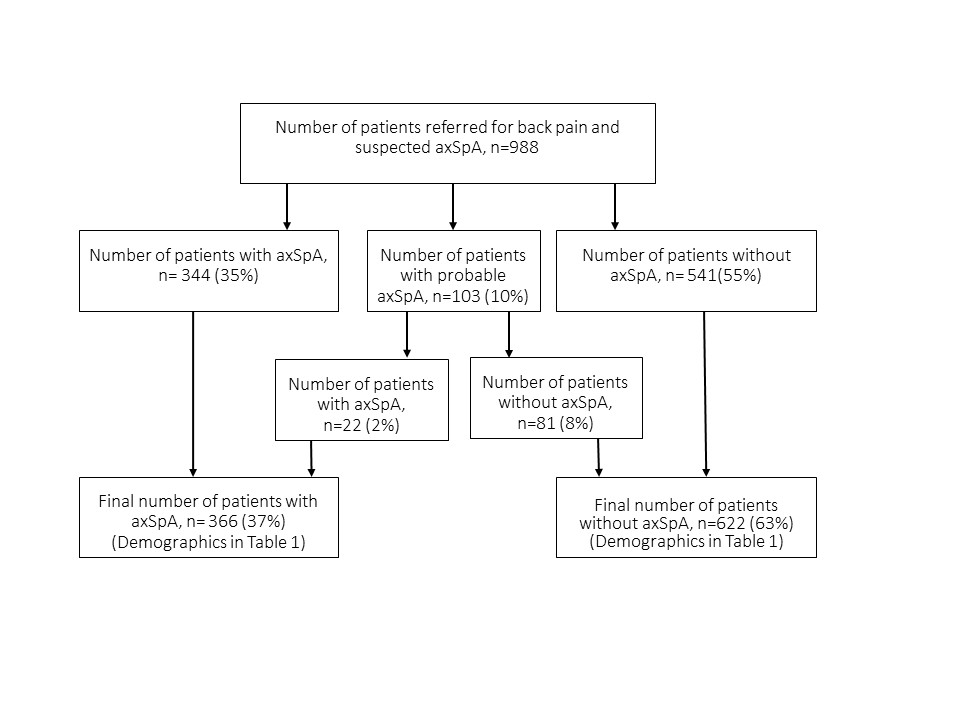

Supplement: rkae102_Supplementary_Data [file rkae102_supplementary_data.docx]
